# Supplementary material for: LSD-induced increase of Ising temperature and algorithmic complexity of brain dynamics
Source: PLoS Comput Biol. 2023 Feb 3;19(2):e1010811. doi: 10.1371/journal.pcbi.1010811 (PMC9943020; doi:10.1371/journal.pcbi.1010811)
Supplement: S1 Text — This file contains figures and tables in the following sections: 1) List of AAL areas, 2) Changes in connectivity, 3) Susceptibility per parcel, 4) Empirical FC, 5) Ising and dMRI connectomes, 6) Network plots, 7) Group FC for each condition (empirical vs. Ising), 8) Homotopic FC and FC negativity. FC for each subject and condition, 9) Homotopic and link susceptibility, 10) Correlation between metrics, 11) Subject questionnaire scores and their correlation with metrics. (PDF) [file pcbi.1010811.s001.pdf]

S1 - Supporting Information

For: *LSD-induced increase of Ising temperature and algorithmic complexity of brain dynamics* (2022/23), Giulio Ruffini, Giada Damiani, Diego Lozano-Soldevilla, Nikolas Deco, Fernando E. Rosas, Narsis A. Kiani, Adri án Ponce-Alvarez10, Morten L. Kringelbach, Robin Carhart-Harris, Gustavo Deco, Plos Computational Biology.

Contents

1 List of AAL areas **2**

2 Changes in homotopic connectivity **3**

3 Susceptibility per parcel **4**

4 Empirical FC: all data and change between conditions **5**

5 Ising and dMRI connectome comparison (detailed view) **7**

6 Network plots **9**

7 Group FC for each condition (empirical vs. Ising) **10**

8 Homotopic FC and FC negativity. FC for each subject and condition. **11**

9 Homotopic and link susceptibility **13**

9.1 Impact of decreased homotopic connectivity 13

9.2 Link susceptibility 13

10 Scatterplots and relation of metrics **15**

11 Subjects questionnaire scores and their correlation with metrics **16**

1 List of AAL areas

Here we report the list of AAL areas in the specific order they were used in this study (Table A).

Table A. Regions of interest from AAL atlas. The AAL areas were ordered in the following way: first all the areas in the left hemisphere (LH), and then all the areas in the right hemisphere (RH) in reverse order, so that they are ”symmetrized” with respect to the contralateral hemisphere.

idx	ROI name		idx	ROI name	
1	Precentral	LH	46	Temporal_Inf	RH
2	Frontal_Sup	LH	47	Temp_Pole_Mid	RH
3	Frontal_Sup_Orb	LH	48	Temporal_Mid	RH
4	Frontal_Mid	LH	49	Temp_Pole_Sup	RH
5	Frontal_Mid_Orb	LH	50	Temporal_Sup	RH
6	Frontal_Inf_Oper	LH	51	Heschl	RH
7	Frontal_Inf_Tri	LH	52	Thalamus	RH
8	Frontal_Inf_Orb	LH	53	Pallidum	RH
9	Rolandic_Oper	LH	54	Putamen	RH
10	Supp_Motor	LH	55	Caudate	RH
11	Olfactory	LH	56	Paracentral_Lob	RH
12	Frontal_Sup_Med	LH	57	Precuneus	RH
13	Frontal_Med_Orb	LH	58	Angular	RH
14	Rectus	LH	59	SupraMarginal	RH
15	Insula	LH	60	Parietal_Inf	RH
16	Cingulum_Ant	LH	61	Parietal_Sup	RH
17	Cingulum_Mid	LH	62	Postcentral	RH
18	Cingulum_Post	LH	63	Fusiform	RH
19	Hippocampus	LH	64	Occipital_Inf	RH
20	ParaHippo	LH	65	Occipital_Mid	RH
21	Amygdala	LH	66	Occipital_Sup	RH
22	Calcarine	LH	67	Lingual	RH
23	Cuneus	LH	68	Cuneus	RH
24	Lingual	LH	69	Calcarine	RH
25	Occipital_Sup	LH	70	Amygdala	RH
26	Occipital_Mid	LH	71	ParaHippo	RH
27	Occipital_Inf	LH	72	Hippocampus	RH
28	Fusiform	LH	73	Cingulum_Post	RH
29	Postcentral	LH	74	Cingulum_Mid	RH
30	Parietal_Sup	LH	75	Cingulum_Ant	RH
31	Parietal_Inf	LH	76	Insula	RH
32	SupraMarginal	LH	77	Rectus	RH
33	Angular	LH	78	Frontal_Med_Orb	RH
34	Precuneus	LH	79	Frontal_Sup_Med	RH
35	Paracentral_Lob	LH	80	Olfactory	RH
36	Caudate	LH	81	Supp_Motor	RH
37	Putamen	LH	82	Rolandic_Oper	RH
38	Pallidum	LH	83	Frontal_Inf_Orb	RH
39	Thalamus	LH	84	Frontal_Inf_Tri	RH
40	Heschl	LH	85	Frontal_Inf_Oper	RH
41	Temporal_Sup	LH	86	Frontal_Mid_Orb	RH
42	Temp_Pole_Sup	LH	87	Frontal_Mid	RH
43	Temporal_Mid	LH	88	Frontal_Sup_Orb	RH
44	Temp_Pole_Mid	LH	89	Frontal_Sup	RH
45	Temporal_Inf	LH	90	Precentral	RH

2 Changes in homotopic connectivity

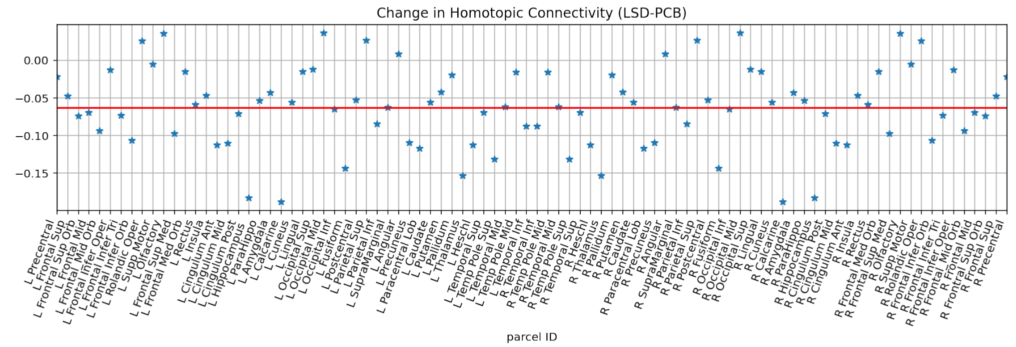

Fig A. Changes in homotopic connectivity in Ising connectivity matrix (LSD-placebo).
The largest decreases under LSD can be observed in the Calcarine (ID 19), Hippocampus (22), Thalamus (39), and Fusiform (28) areas. The largest increases are found in the Rolandic Operculum (9), Olfactory (11), Occipital Mid (26), and Parietal Sup (30) areas. The red line indicates the mean across all points (negative in this case, since there is an average decrease in homotopic connectivity).

3 Susceptibility per parcel

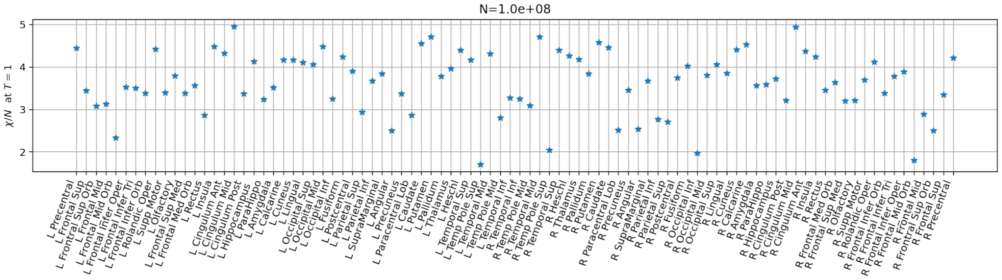

Fig B. Susceptibility per parcel at $T = 1$.
For each parcel, the susceptibility of each parcel at the nominal $T = 1$ temperature of the global archetype Ising model is provided.

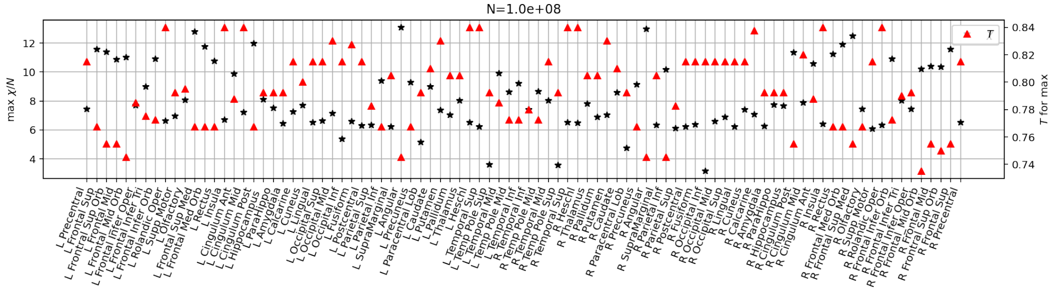

Fig C. Maximum susceptibility per parcel and corresponding temperature.
For each parcel, the maximum susceptibility (black asterisk) and the temperature at which it is attained (red triangle) are provided for the global Ising archetype model.

4 Empirical FC: all data and change between conditions

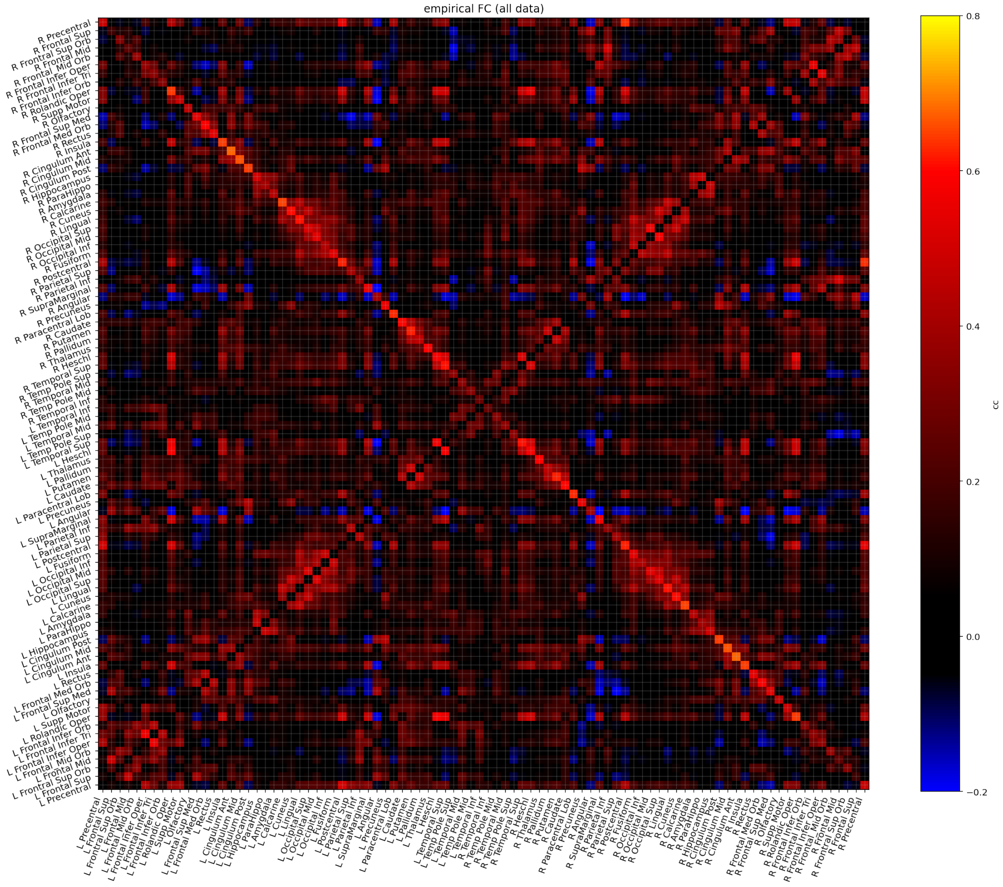

Fig D. Empirical FC, all data.
Pearson correlation between the binarized average activity of each parcel.

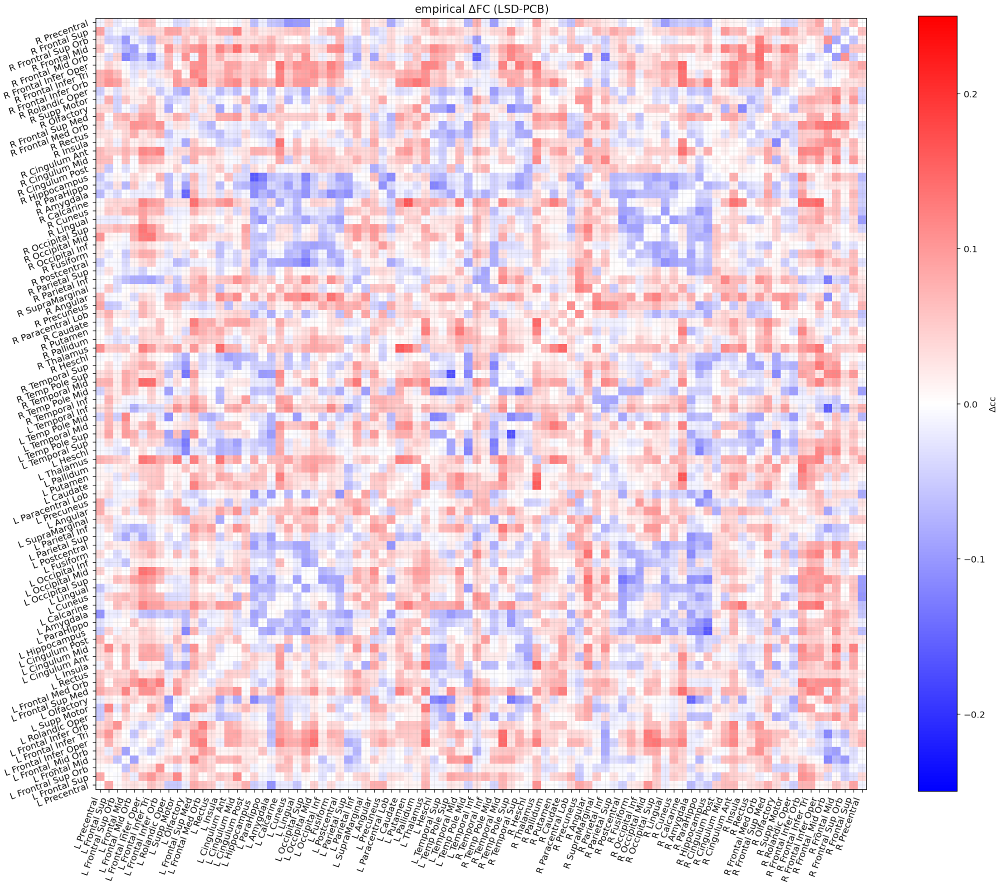

Fig E. Change in empirical FC between two conditions (LSD minus placebo). Change in the functional connectivity (defined by Pearson correlation of the averaged activity in each parcel) computed from the concatenated binarized data in each condition.

5 Ising and dMRI connectome comparison (detailed view)

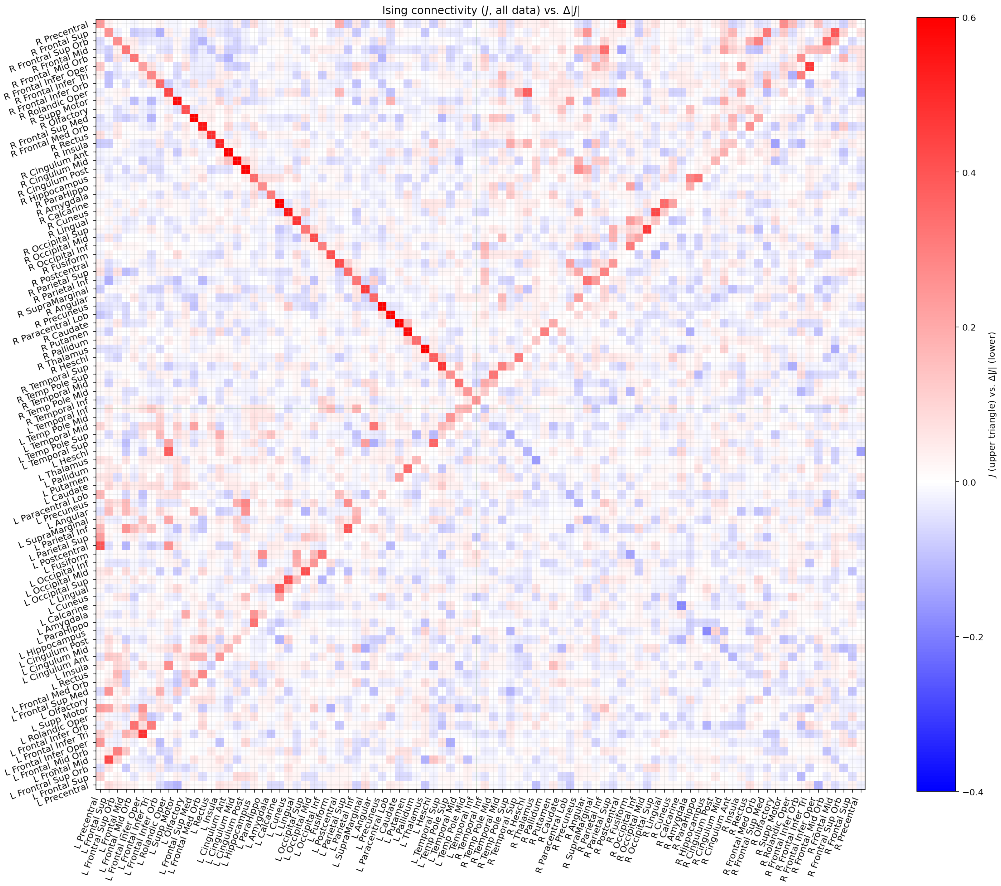

Fig F. Global archetype connectivity and its change across conditions. Global archetype (combining all data) Ising connectivity J (upper left triangle), and the change in link strength ($\Delta|J|$, LSD-placebo) (lower right triangle). The latter is obtained by creating separate archetypes from all the data in the LSD and placebo conditions. The down-trending diagonal values reflect strong inter-hemispheric homotopic connectivity and its change (decrease in LSD condition). By construction, the connectomes have zeros in the diagonal ($J_{ii} = 0$).

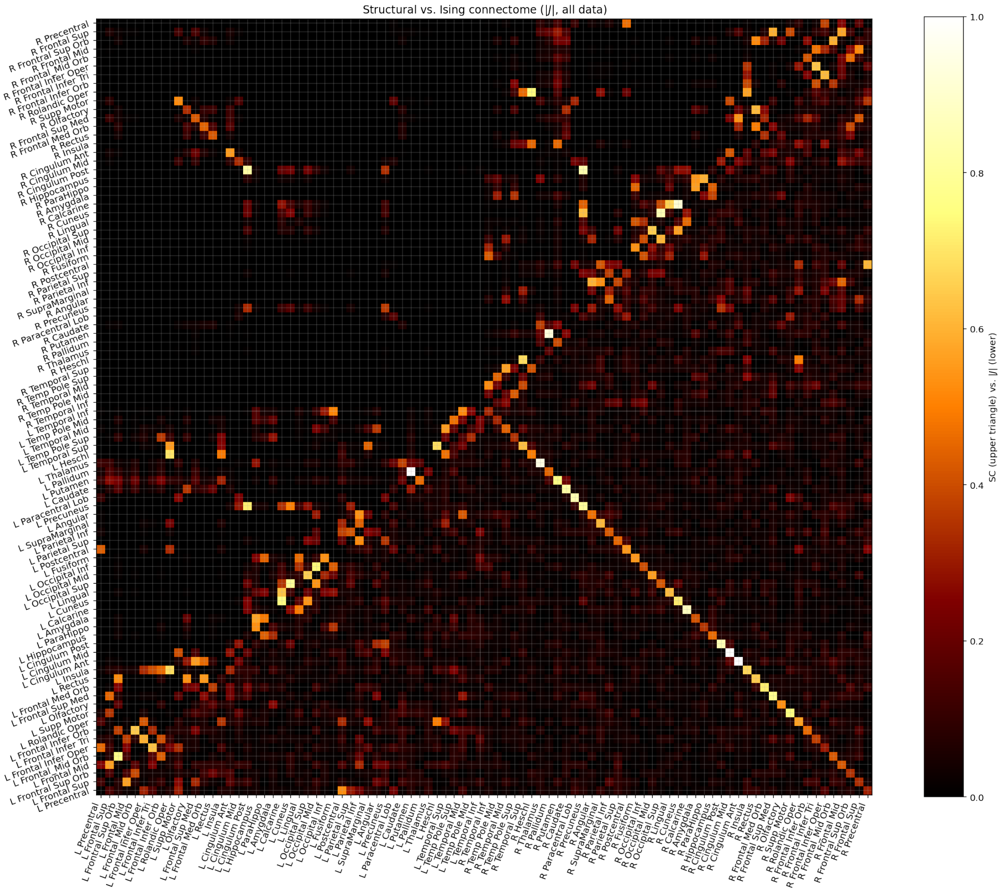

Fig G. Ising-derived connectome.
AAL structural connectome obtained from dMRI (upper left triangle) and unsigned global archetype Ising connectivity (lower right triangle). The most important difference is in the increased homotopic connectivity in the Ising connectome.

6 Network plots

Here we provide plots of the Ising connectivity magnitude change (LSD-PCBO).

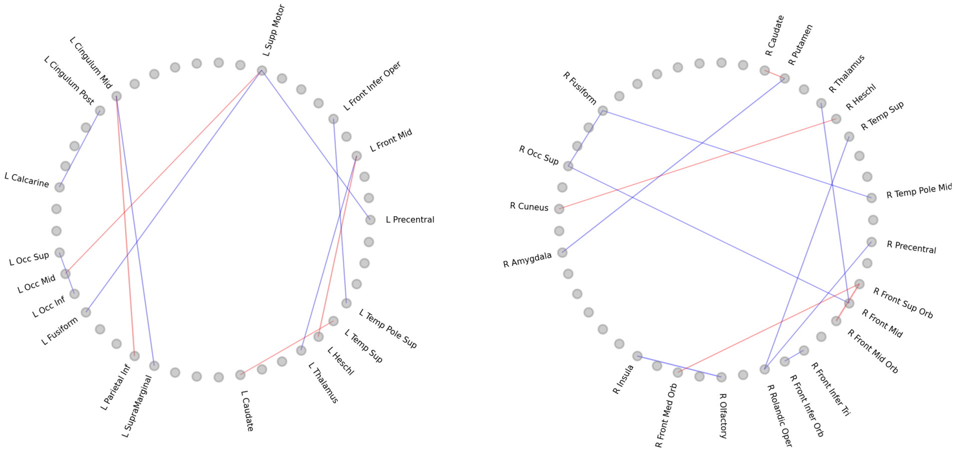

Fig H. Intra-hemispheric connectivity changes under LSD.
Intra-hemispheric network plots highlighting changes ($\Delta|J|$, LSD-PCBO) higher than 0.11 in the left (on the left) and right (right) hemispheres. Red (blue) = positive (negative) connection changes. That is, a blue link indicates a loss of connectivity under LSD w.r.t. placebo.

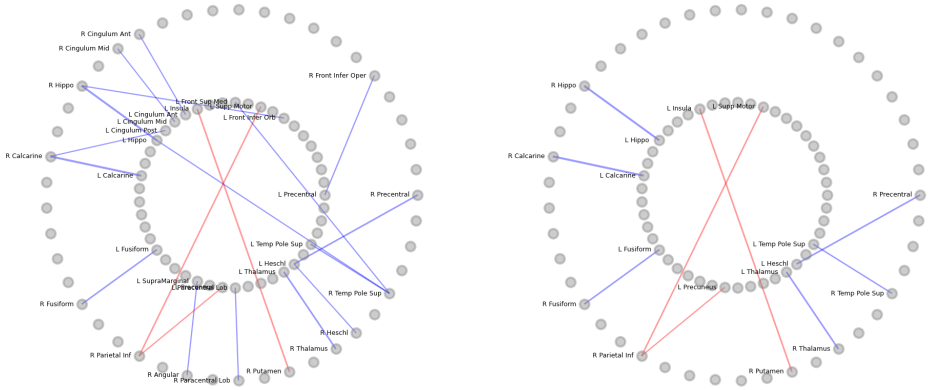

Fig I. Inter-hemispheric connectivity changes under LSD.
Inter-hemispheric network plots highlighting changes ($\Delta|J|$, LSD-PCBO) higher than 0.11 (left) and 0.12 (right). The inner (outer) circle displays the left (right) hemisphere nodes. Red (blue) = positive (negative) connection changes.

7 Group FC for each condition (empirical vs. Ising)

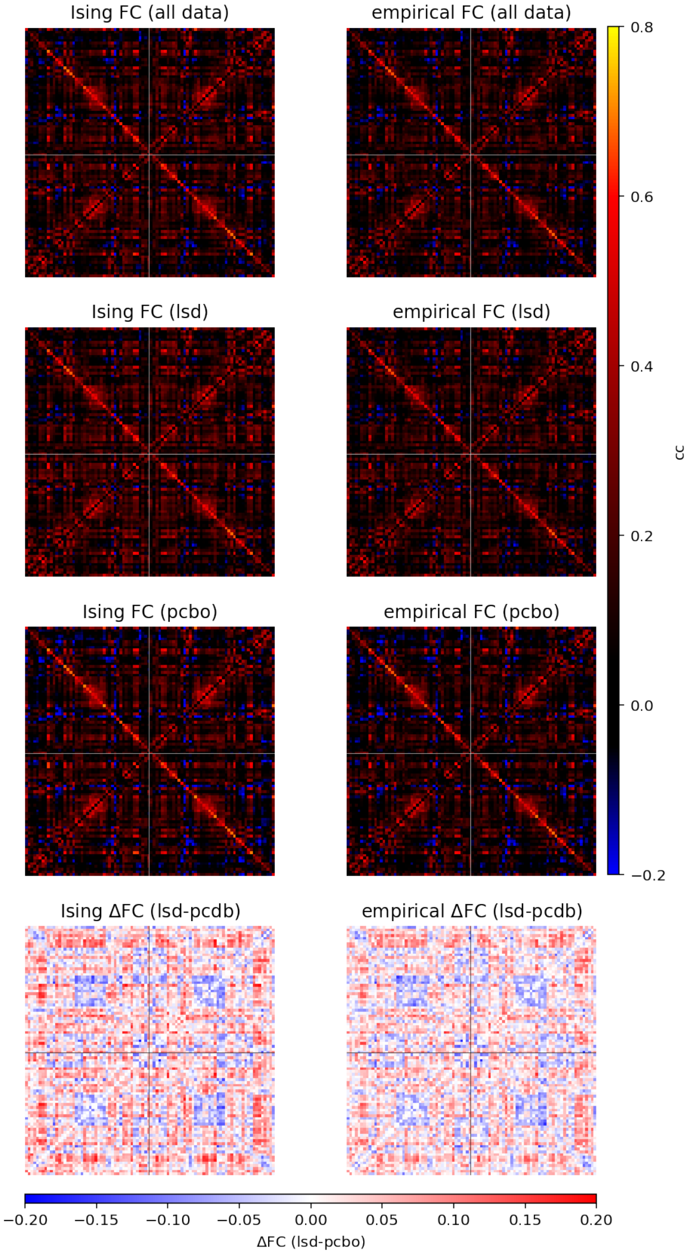

Fig J. FC for each condition, empirical vs. Ising generated (LSD, placebo and difference).
Left column: FC generated by each archetype (LSD and placebo) and difference in the bottom. We note that 1 bit quantization produces a loss of correlation with respect to unbinarized correlation. **Right column:** Empirical FC for each condition and difference (bottom) from binarized data. The Pearson correlation between the empirical and Ising FC (all data) is 0.99, and between the difference (delta FC of LSD-placebo, empirical vs. Ising) is 0.96.

8 Homotopic FC and FC negativity. FC for each subject and condition.

Fig K provides boxplots of the averaged homotopic FC (average of the FC of a subset of homotopic links) and the negativity of each FC matrix. We define negativity by the percent weight of negative weights,

$$\eta = 100 \cdot \frac{\sum_{ij} |FC_{ij}| - \sum_{ij} FC_{ij}}{2 \sum_{ij} |FC_{ij}|} \tag{1}$$

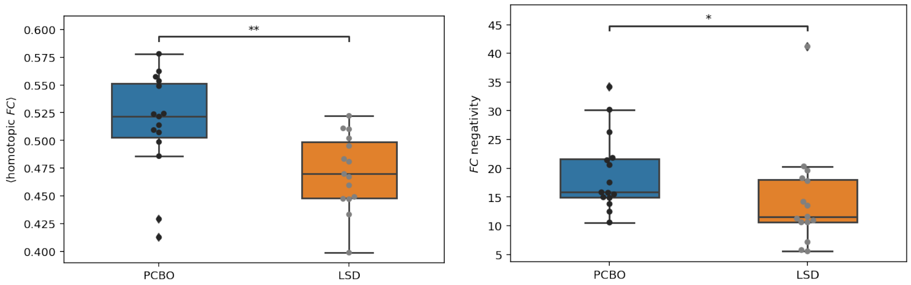

Fig K. Homotopic FC for the two groups and percent of weight of negative values in FC (negativity).
Left: Homotopic connectivity (paired Wilcoxon statistic=10.0, p=0.003). **Right:** FC negativity (paired Wilcoxon statistic=16.0, p=0.010)

In the next figures, we provide the FC data for each subject (Fig L).

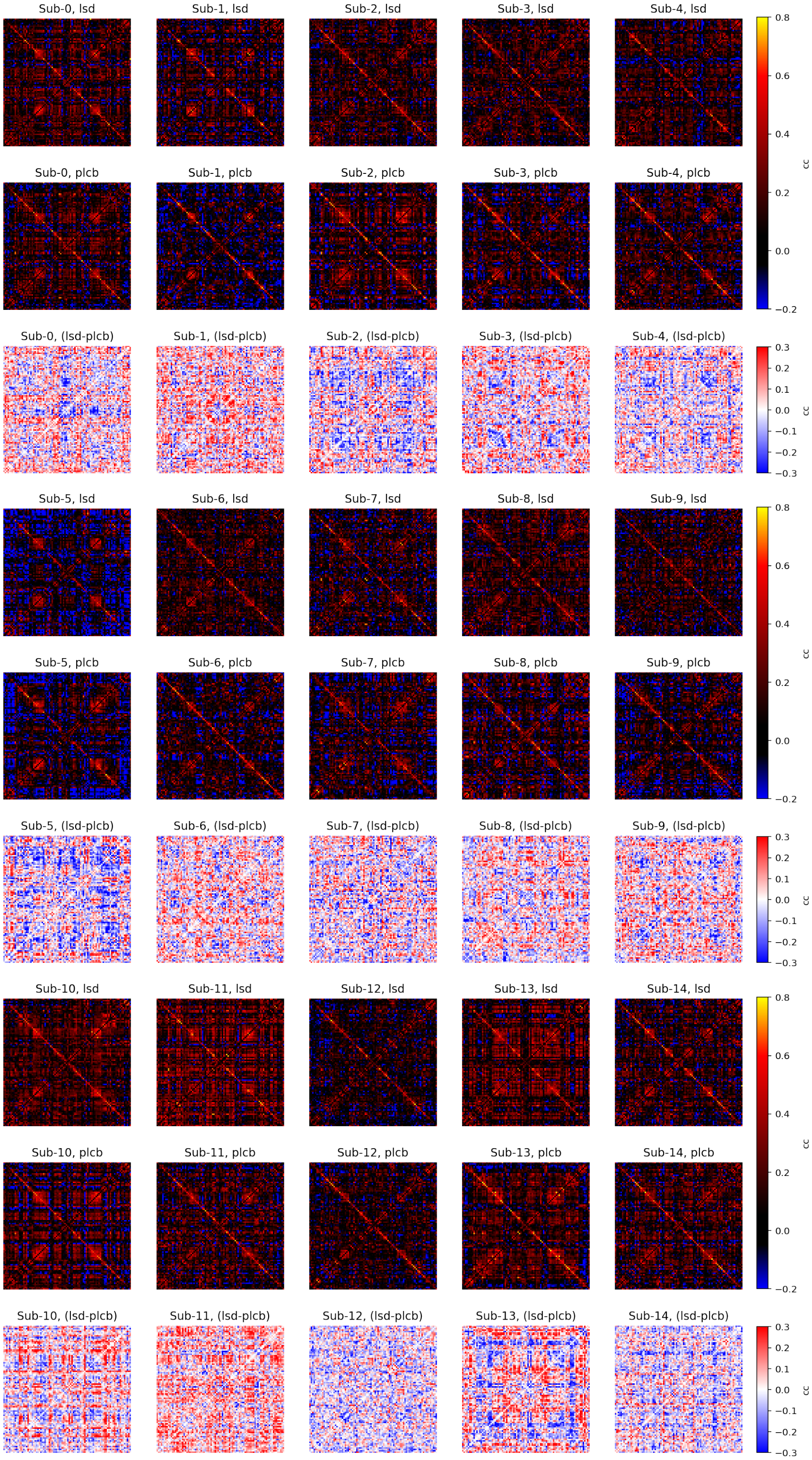

Fig L. FC of binarized empirical data for each subject and condition, and their difference. The color scale is adjusted to display weaker but numerous negative links in FC.

9 Homotopic and link susceptibility

9.1 Impact of decreased homotopic connectivity

Fig M provides plots of susceptibility for the placebo data archetype and the effect of reducing homotopic connectivity by 20%, which decreases the critical temperature and leads to loss of FC. Decreasing the critical temperature means that the system at the nominal temperature of $T = 1$ is more disordered.

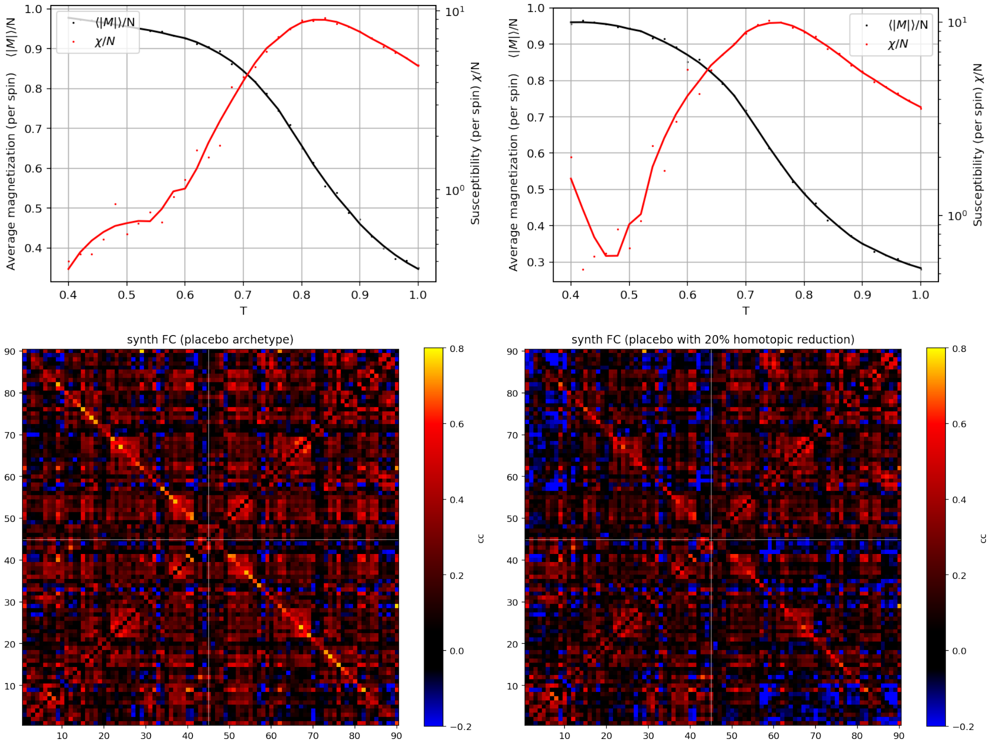

Fig M. Impact of reduced homotopic links strength:
Top left: Susceptibility and magnetization plot of placebo archetype ($T_c = 0.84$). **Top Right:** susceptibility and magnetization plot after reducing homotopic link strength in the archetype by 20% ($T_c = 0.74$). **Bottom row:** FC of synthetic data generated by each model at $T = 1$ (the nominal system temperature of the archetype).

9.2 Link susceptibility

Next, we analyze the concept of link susceptibility. The link susceptibility

$$l_{ij} = \frac{\partial \langle M \rangle}{\partial J_{ij}}\bigg|_T = \beta \left(\langle M \sigma_i \sigma_j \rangle - \langle M \rangle \langle \sigma_i \sigma_j \rangle \right) \tag{2}$$

provides information about the sensitivity to additive changes to a particular link in the system. To understand the impact of changes in the scale of a particular link ($J \rightarrow J + \alpha J$), we use the scaled quantity $L_{ij} = l_{ij} J_{ij}$, which provides a metric on the susceptibility of the mean magnetization of the model to multiplicative changes in archetype link strength,

$$\delta \langle M \rangle = L_{ij} \delta \alpha \tag{3}$$

Fig N provides the link and scaled link susceptibility for the placebo archetype (i.e., archetype generated by concatenating all the binarized subject data in the placebo condition) near the critical temperature.

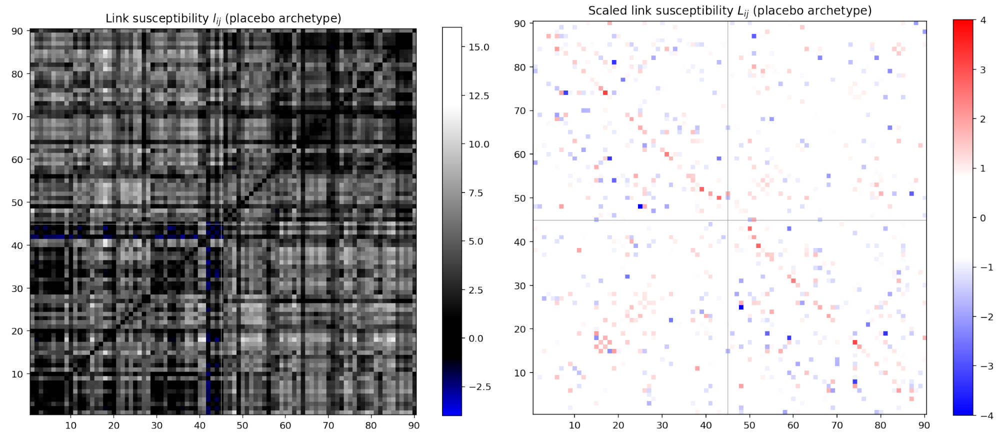

Fig N. Link susceptibility of the placebo archetype.
Left: link susceptibility plot of placebo archetype. **Right:** scaled link susceptibility.

10 Scatterplots and relation of metrics

We provide scatter plots of features extracted from data as shown in Figure O. For statistics see tables in main text.

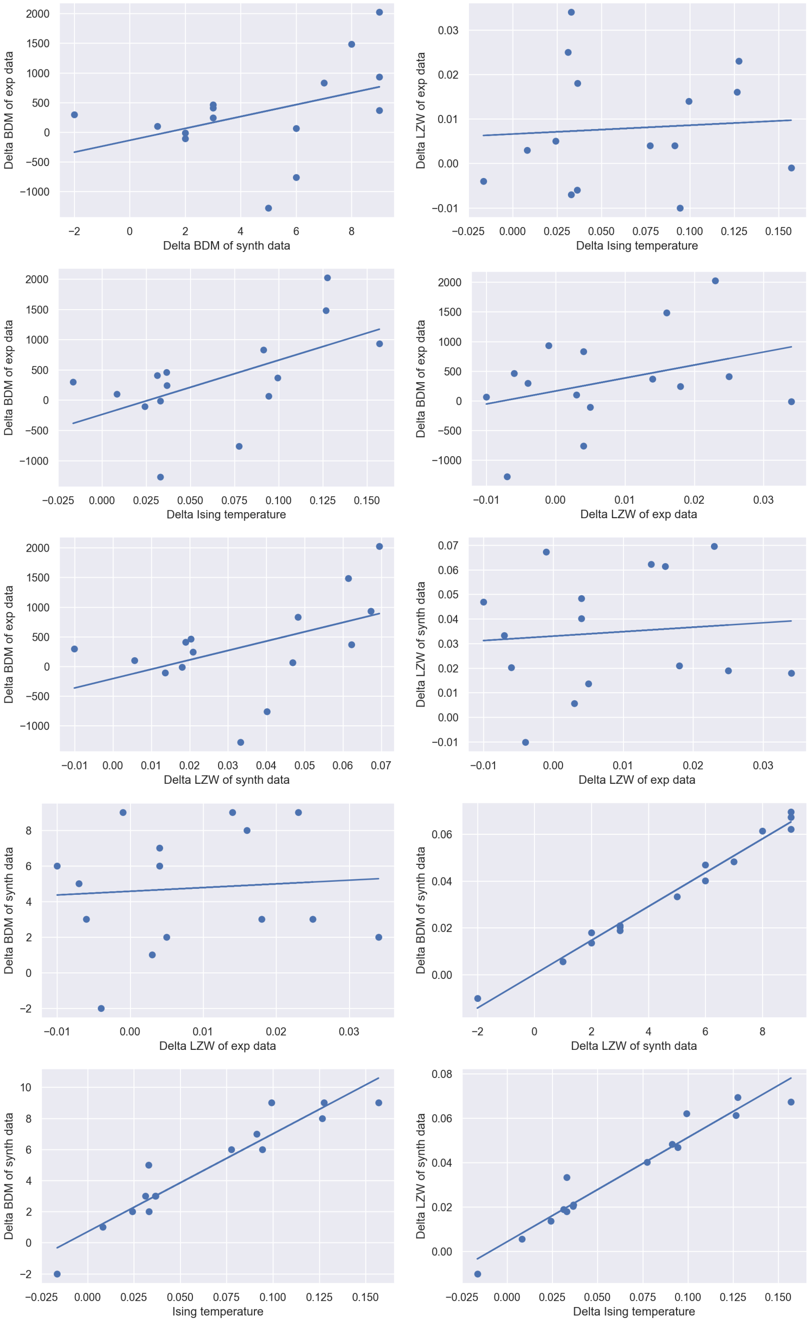

Fig O. Correlation scatter plots between the metrics. Correlation plots between the metrics, i.e., delta LZW complexity of the simulated and empirical data, delta Ising temperature, and delta BDM complexity of the simulated and empirical data.

11 Subjects questionnaire scores and their correlation with metrics

Difference between LSD and placebo												
Subjects	Unity	Spirit	Bliss	Insight	Disem	Cont&Cog	Anxiety	Complex	Element	Aud/Vis	Meaning Perc	Average
1	0.0	0.0	0.0	0.0	0.1	0.0	0.0	0.1	1.0	0.3	0.0	0.1
2	0.4	0.2	0.4	0.4	0.5	0.2	0.0	0.6	0.6	0.7	0.3	0.4
3	0.0	0.2	0.1	0.3	0.0	0.2	0.1	0.1	0.2	0.2	0.2	0.1
4	0.8	0.3	1.0	0.8	1.0	0.6	0.5	0.7	1.0	0.9	0.5	0.7
5	0.0	0.0	0.0	0.0	0.0	0.0	0.0	0.0	0.0	0.0	0.0	0.0
6	0.2	0.0	0.3	0.5	0.7	0.0	0.0	0.5	1.0	0.7	0.5	0.4
7	0.2	0.1	0.1	0.1	0.7	0.6	0.3	0.4	1.0	0.0	0.2	0.3
8	0.1	0.2	0.2	0.1	0.0	0.1	0.0	0.2	0.6	0.1	0.0	0.1
9	0.2	0.0	0.0	0.0	0.0	0.1	0.0	0.5	1.0	1.0	0.0	0.2
10	0.2	0.3	0.2	0.5	0.0	0.2	0.3	0.8	0.9	0.9	0.3	0.4
11	0.2	0.1	0.3	0.2	0.5	0.3	0.1	0.4	0.6	0.3	0.2	0.3
12	0.7	0.6	0.5	0.3	0.3	0.0	-0.1	0.5	0.2	0.0	0.7	0.3
13	0.4	0.3	0.3	0.2	0.3	0.2	0.0	0.2	0.5	0.6	0.5	0.3
14	0.4	0.3	0.3	0.3	0.5	0.5	0.2	1.0	1.0	1.0	0.9	0.6
15	0.5	0.5	0.6	0.5	0.3	0.4	0.1	0.5	0.4	0.9	0.7	0.5
Ising cc	-0.07	0.06	-0.21	-0.10	-0.10	0.23	0.26	0.42	0.19	0.27	0.11	0.14
LZW cc	-0.13	-0.37	0.00	0.00	0.19	0.26	0.38	0.03	0.48	0.01	-0.10	0.11
BDM cc	-0.02	-0.03	-0.09	-0.10	0.20	0.38	0.40	0.34	0.17	-0.19	0.17	0.13

Table B. ASC questionnaire scores and their correlation with features extracted from data. The higher scores are colored in darker yellow, whereas Pearson correlation coefficients higher in absolute value than 0.30 are colored in blue. Full names of ASC questionnaire categories: Experience of unity, Spiritual experience, Blissful state, Insightfulness, Disembodiment, Impaired control and cognition, Anxiety, Complex imagery, Elementary imagery, Audio-visual synesthesiae, Changed meaning of percepts.

Average difference between LSD and placebo for session 1 and 2							
Subject	Intensity	Simple	Complex	Emotional Arousal	Positive Mood	Ego Dissolution	Average
1	11	16	11	3	10	0	8
2	12	10	7	12	4	16	10
3	11	5	1	3	2	0	3
4	13	20	20	11	10	2	13
5	9	0	1	12	-1	0	4
6	15	17	17	6	10	6	12
7	11	1	1	12	0	1	4
8	9	6	3	10	1	0	5
9	4	4	0	5	7	0	3
10	13	11	3	10	0	0	6
11	11	9	10	10	12	3	9
12	19	15	12	19	19	20	17
13	15	10	4	15	5	11	10
14	19	19	19	18	17	17	18
15	15	5	6	14	8	11	10
Ising cc	0.10	-0.15	-0.26	0.31	-0.33	0.11	-0.07
LZW cc	-0.07	0.34	0.35	-0.38	0.06	-0.22	0.05
BDM cc	0.31	0.15	0.19	0.39	0.02	0.11	0.22

Table C. VAS questionnaire scores and their correlation with features extracted from data. The higher scores are colored in darker yellow, whereas Pearson correlation coefficients higher in absolute value than 0.30 are colored in blue.
